# A genome-wide scan of wastewater *E. coli* for genes under positive selection: focusing on mechanisms of antibiotic resistance

**DOI:** 10.1038/s41598-022-11432-0

**Published:** 2022-05-16

**Authors:** Negin Malekian, Amay A. Agrawal, Thomas U. Berendonk, Ali Al-Fatlawi, Michael Schroeder

**Affiliations:** 1 Biotechnology Center (BIOTEC), Technische Universität Dresden, Tatzberg 47-49, 01307 Dresden, Germany; 2 Institute of Hydrobiology, Technische Universität Dresden, 01217 Dresden, Germany

**Keywords:** Bacterial evolution, Bacterial genomics

## Abstract

Antibiotic resistance is a global health threat and consequently, there is a need to understand the mechanisms driving its emergence. Here, we hypothesize that genes and mutations under positive selection may contribute to antibiotic resistance. We explored wastewater *E. coli*, whose genomes are highly diverse. We subjected 92 genomes to a statistical analysis for positively selected genes. We obtained 75 genes under positive selection and explored their potential for antibiotic resistance. We found that eight genes have functions relating to antibiotic resistance, such as biofilm formation, membrane permeability, and bacterial persistence. Finally, we correlated the presence/absence of non-synonymous mutations in positively selected sites of the genes with a function in resistance against 20 most prescribed antibiotics. We identified mutations associated with antibiotic resistance in two genes: the porin *ompC* and the bacterial persistence gene *hipA*. These mutations are located at the surface of the proteins and may hence have a direct effect on structure and function. For *hipA*, we hypothesize that the mutations influence its interaction with *hipB* and that they enhance the capacity for dormancy as a strategy to evade antibiotics. Overall, genomic data and positive selection analyses uncover novel insights into mechanisms driving antibiotic resistance.

## Introduction

“The time may come when antibiotics can be bought by anyone in the shops. Then there is the danger that the ignorant man may easily underdose himself, and by exposing his microbes to non-lethal quantities of the drug, make them resistant.”, warned Alexander Fleming, the inventor of the world's first antibiotic, during his Nobel lecture^1^ about the evolution of antibiotic resistance via positive selection. In fact, bacteria with a beneficial mutation for antibiotic resistance tend to survive and reproduce more than other bacteria, resulting in the development of a resistant population. Now, one century after the discovery of the first antibiotic, antibiotic resistance is on the rise across the world. Antibiotic therapy failures cause hundreds of thousands of deaths each year, posing a growing threat to public health^2^. Thus, having a deep insight into the evolution of antibiotic resistance and the genomics behind it might aid in the development of effective treatments that are less likely to evolve resistance^2^.

The advances in DNA sequencing technologies have provided us with the opportunity to scan genomes very fast and cheaply. Using sequencing data, one can find the selection footprints in genomes. One approach to determining selection footprints compares the amount of synonymous and non-synonymous mutations within a gene. The assumption is that synonymous mutations do not change the amino acid sequence and hence do not have a direct effect on the gene product. In contrast, non-synonymous mutations alter the gene product and might therefore confer a change in function, which represents an adaptation to an environmental change. Under randomness, one expects the amount of synonymous and non-synonymous mutations to be the same on average, but a selective pressure (i.e. any pressure affecting the survival or reproductive success in a portion of a population) may make them differ. This intuition is captured by the ratio ω of non-synonymous to synonymous mutations within a gene or a region within a gene^3^. An ω value of greater than one means that the gene or region is under positive selection. Similarly, ω less than one indicates negative selection. A model that allows the variation of ω in different parts of genes is preferred as not all regions inside the genes are under positive selection^4^.

Generally, the assumption that synonymous mutations do not contribute to adaptation processes may not always be valid. It has been shown that synonymous mutations can indirectly impact function by changing gene expression, gene regulation, and other cellular processes^2^. For example, in an epistasis process, a trait may be influenced by a possible interaction between mutations in the same or different genes^5,6^. Consequently, non-significant or synonymous mutations that interact with each other could also have a considerable influence. As another example, mutations can influence the activity of other genes via epigenetic modification^7^. However, in the scope of this work, since the effect of synonymous mutations is indirect and difficult to measure and quantify, most previous studies on positive selection in bacteria rely on the ratio of substitution rate.

The previous studies of positive selection in *E. coli*^8–10^ analysed a small number of genomes. Chen et al.^8^ investigated seven *E. coli* genomes, Petersen et al.^9^ six *E. coli* and *Shigella flexneri* genomes, and Rojas et al. eight avian pathogenic *E. coli*^10^ genomes. They all showed that positive selection in wild-type *E. coli* targets a wide range of different functions. None of these studies focused on positive selection in the context of antibiotic resistance.

**Figure 1:**
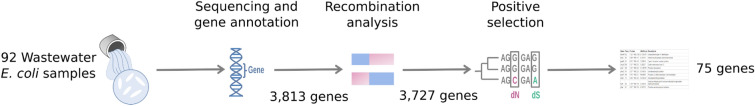
Overview of our study. We collected 92 *E. coli* genomes from a wastewater treatment plant in Dresden, Germany. We filtered genes under recombination and analyzed the remaining genes for positive selection.

Here, we address this open problem. We go beyond these previous studies by considering a larger data set of 92 wild-type genomes of *E. coli* and by ensuring maximal diversity of genomes. We achieve the latter by investigating genomes originating from a wastewater treatment plant (WWTP). Wastewater treatment plants are melting pots for bacteria of human, clinical, animal, and environmental origin harboring highly diverse *E. coli* genomes^11,12^. These diverse sources furnish wastewater with various chemical pollutants such as antibiotics, excreted by humans and animals, or improper medication disposal methods, causing selective pressure in this environment^13^. Similar to the previous studies of positive selection in *E. coli*, we will apply the substitution rate model to pinpoint genes under positive selection and will discuss their function. We expect that genes with important functions are core genes, i.e. present in nearly all genomes. Furthermore, we hypothesise that positive selection in a wastewater treatment plant will favour genes related to antibiotic resistance, and therefore the presence/absence of non-synonymous mutations in positively selected sites of these genes will be correlated with our antibiotic resistance values. An overview of our study can be seen in Fig. 1.

## Methods

### Sequence data and resistance labels

We used 92 *E. coli* genome sequences^11^ (available from NCBI’s assembly database, PRJNA380388: https://www.ncbi.nlm.nih.gov/assembly/?term=PRJNA380388). We collected this data from the inflow and outflow of the municipal wastewater treatment plant in Dresden, Germany. The antibiotic resistance phenotype was measured by using the agar disk diffusion method. More details regarding the sequence data and their resistance phenotype are available in^11^.

### Multiple sequence alignment and phylogenetic tree

 We aligned the protein sequences for 3823 genes in different isolates which are in common with *E. coli* k12 (our reference genome) using Clustal Omega^14^. However, the results of Clustal Omega were not always matching the input format of the tool for detecting positive selection (PAML^3^), i.e. the sequence length was not a multiple of three. In these cases, we used the MUSCLE algorithm^15^ for the alignment. For ten genes, however, their multiple sequence alignment (MSA) created by either method, was not suitable for the PAML tool. Thus, we removed them from the downstream analysis and ran the downstream analysis with 3813 genes. To generate phylogenetic trees based on the MSA of all the genes, we used the “Maximum Likelihood” method implemented in the MEGA software^16^.

### Recombination test

To reduce the risk of falsely increased substitution rates, we applied a recombination test. We used the single recombination breakpoint (SBP) method of the HyPhy software^17^ to detect the genes under recombination. This technique relies on the fact that, under recombination, a single phylogenetic tree cannot describe the whole alignment. Instead, multiple phylogenetic trees are required to represent different parts of the alignment^18^. After conducting the recombination test, 86 genes that showed evidence of recombination were excluded from our further analysis. Finally, the positive selection analysis was performed on the remaining 3727 genes.

### Test for positive selection

We used the site model in the PAML tool^3^ to detect the genes under positive selection. This model uses the ratio of non-synonymous (dN) to synonymous (dS) substitutions (also called ratio of substitution rate: ω = dN/dS), where the ω value can vary among different codon sites inside the genes. This tool applies the maximum likelihood algorithm to assign scores to different selection models, providing users with a measurement to choose the best model describing their data. In our case, we compared a model of positive selection (M2a) against a model of neutral selection (M1a: the null hypothesis). To avoid the problem of multiple testing, we calculated a Bonferroni-corrected *p* value threshold based on the significance level of 0.05. To do so, we divided the significance level of 0.05 by the total number of genes (0.05/3727 = 1.34 × 10E−5).

### Gene function, essentiality, and conservation analysis

We functionally annotated and categorized the genes with the evidence of positive selection into different groups using the EcoCyc database^19^. In addition, we examined the literature and delved further into the function of these genes, especially in relation to antibiotic resistance. We checked the essentiality of our genes in the following datasets: Gerdes et al. dataset^20^, the Keio collection^21^, and the Profiling *E. coli* Chromosome (PEC) database^22^. Also, we defined the genes with a frequency of more than 95% among genomes as core-genome and considered them conserved. The same was applied for defining conserved core mutations.

### Structure analysis

For the positively selected core genes with the evidence of antibiotic resistance, we downloaded the 3D protein structures from PDB^23^ (OmpC: 2j1N, OmpA: 1qjP, HipA: 3TPD) or the Alphafold database^24^ if no PDB structure was available (DgcE, YfaL, and YdhQ). Structures were visualised with Pymol 2.2.0. We highlighted the positively selected sites with posterior probability *P* > *95%*.

### Antibiotic resistance

For the positively selected core genes with the evidence of antibiotic resistance, we checked the possible association between their positively selected sites and resistance against 20 widely used antibiotics covering all major classes of antibiotics (for details see^11,42^). We built a linear regression model between continuous values of antibiotic resistance (diameter of inhibition zone in disk diffusion method) and the presence/absence of non-synonymous variants in positively selected sites and reported the sites of non-synonymous mutations which are positively correlated with antibiotic resistance. To do so, we used the linregress method from the stats package in Python.

## Results

We analysed 92 *E. coli* genomes from a wastewater treatment plant and subjected them to a positive selection analysis by ratios of substitution rates as implemented in the PAML tool^3^. From the total of 3813 distinct genes across the 92 genomes, we filtered out 86 genes with evidence for recombination (see “Methods” section) and consequently falsely increased substitution rates. From the remaining 3727 genes, we identified 75 significant genes for positive selection, which passed the Bonferroni-corrected *p* value of 1.34 × 10E−5 (Supplementary Table S1). The top gene, *hemX* stands out with a highly significant *p* value, over 100 orders of magnitude better than the imposed threshold. Generallythe top 14 genes (*hemX*, *hisC*, *gspK*, *ompC*, *ydfJ*, *yhaC*, *paaF*, *elaD*, *folK*,* yfaL*, *ycgV*,* lacZ*, *rhsC*, and *flgK*) have a *p* value better than 10E−15 underlining the strength of the statistical signal.

We proceed in three steps. First, we validated the identified genes against those known from the literature. We expected some overlap, but as we have a much larger data set than previous studies, we expected a number of new genes. We characterised the function of these genes and focused in particular on antibiotic resistance mechanisms. Second, we reasoned that genes with important functions consistently appear in nearly all genomes, i.e. they are so called core genes. Moreover, for these core genes it is particularly interesting to answer whether their positive selection is the result of the total mutation load or a single mutation? Third, we zoomed in on the core genes known to be involved in antibiotic resistance. For these, we examined 3D structures to determine whether the mutations are located on the surface of the proteins, where they may play a role in the protein’s function?

### 10% of genes including ompC agree with previous studies

**Figure 2:**
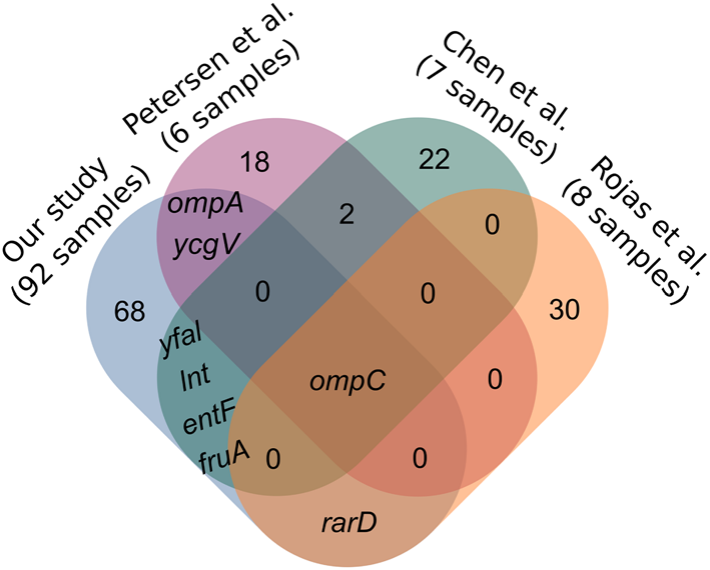
Venn diagram of common genes between previous studies as well as our study. The number in the figure refers to the count of genes. However, for the common genes between our study and previous studies, we showed the name of genes instead of their count. We found eight genes (*ompC*, *ompA*, *ycgV*, *rarD*, *yfaL*, *lnt*, *entF*, and *fruA*) in common with previous studies of positive selection in *E. coli*. The *ompC* gene, which is known to be under positive selection in bacteria, is common to all studies.

While previous studies on positive selection have used far fewer genomes, we nonetheless expected to find some overlap with these data sets. In fact, we found eight genes in common with previous studies on positive selection in *E. coli* (see Fig. 2), namely *ompC*, *yfaL*, *lnt*, *entF*, and *fruA*^8^, *ompC*, *ycgV* and *ompA*^9^, and *ompC* and *rarD*^10^, respectively. Interestingly, *ompC*, which is highly significant in our study with a *p* value of 10E−32, was also found in all three studies.

### Positive selection targets antibiotic resistance

Positive selection targets various functions in bacteria. The genes listed in Table 1a cover a wide variety of functions including antibiotic resistance, DNA damage repair, cell division, production of chemicals. We are particularly interested in the genes that may give resistance to antibiotics and attempt to understand the process by which they might develop resistance (see Table 1b). However, it should be noted that the functions of the genes listed in Table 1b are not only limited to antibiotic resistance but due to the focus of the study, we shed more light on their role in antibiotic resistance. Generally, bacteria have different mechanisms of action to develop resistance against antibiotics, such as increased efflux of antibiotics, limiting uptake of antibiotics, persistence against antibiotics, changing antibiotic targets, and developing biofilm formation^25^. Interestingly, we found that positively selected genes can infiltrate into most of these mechanisms. These positively selected mechanisms of antibiotic resistance are general and independent of the type of antibiotic and antibiotic targets. This was to be anticipated, given that we did not supply any antibiotic resistance labels to our analysis. Among these mechanisms, biofilm formation has the largest number of positively selected genes. In the following, we will shed more light on these mechanisms and the involved genes.

**Table 1:** Targets of positive selection. (a) Generally. (b) With respect to antibiotic resistance.

**(a) Positive selection targets different functions.**	
Function	Genes
Antibiotic resistance	*yfaL*, *ycgV*,* lsrA*, *ydhQ*, *ompC*, *ompA*, *hipA*, *dgcE*
Cellular response to DNA damage stimulus	*btsT*, *yhaC*, *sbcC*, *uxuA*
Cellular protein modification process	*pphC*, *ptsP*, *def*, *ydiU*
Establishment of protein localization to extracellular region	*gspC*, *gspL*, *gspK*, *dtpD*
Carbohydrate transport	*agaC*, *ptsN*, *uidC*, *fruA*
DNA dealkylation involved in DNA repair	*alkA*, *alkB*
DNA restriction-modification system	*dcm*, *hsdS*
tRNA wobble base modification	*tilS*, *tmcA*
Determination of cell shape	*mreC*
Essential for cell division	*ftsK*
Protein deubiquitination	*elaD*
Protein integration into the cell membrane	*ynbB*
Aromatic compounds biosynthesis	*paaF, mhpA, cpdB, deaD*
Folic acid biosynthesis	*folk, abgB*
Glyoxylate biosynthesis	*glcB, icd*
Lactose biosynthesis	*lacZ*
Lipoprotein biosynthesis	*lnt*
Amino acid activation for nonribosomal peptide biosynthesis	*entF*
Uroporphyrinogen III biosynthesis	*hemD*
Glycine betaine biosynthesis	*betA*

**(b) The positively selected genes that are known to be involved in antibiotic resistance have different mechanisms of resistance.**	
Mechanism of antibiotic resistance	Genes
Biofilm formation	*yfaL*, *ycgV*, *lsrA*, *ydhQ*, *dgcE*
Outer membrane permeability	*ompC*, *ompA*
Efflux pumps	*ompA*
Bacterial persistence	*hipA*

### Biofilm formation has the largest number of positively selected genes

Biofilm formation is a known mechanism for bacteria to develop resistance. It is hypothesized that bacteria adhere to different surfaces and form biofilm as part of their adaptation through evolution^26^. In our study, we found five genes that play a vital role in biofilm formation (*yfaL*, *ycgV*, *ydhQ, lsrA,* and *dgcE*). The *yfaL*, *ycgV* and *ydhQ* genes are adhesin-like autotransporter proteins homologous to Antigen 43 (Ag43), a major surface adhesin that promotes cell–cell aggregation and biofilm formation^27^. The induction of *ycgV* and *yfaL* plays an essential role in the development of mature biofilms as it leads to clear adhesion. The *ydhQ*, on the other hand, is required for the early stages of adhesion. It should be noted that bacteria can develop biofilms by diverse mechanisms of action. For example, efflux pumps, which are traditionally known for developing antibiotic resistance by extruding antibiotics from within cells to the out, are recently reported to be involved in biofilm formation^28^. Specifically, the upregulation of transport gene *lsrA* is associated with biofilm growth as it facilitates quorum sensing (QS), a system of intercellular communication required for the adoption and maintenance of a biofilm mode of growth by bacteria. Also, the ubiquitous bacterial second messenger c-di-GMP enhances biofilm formation^29^. The *dgcE* gene that produces c-di-GMP in *E. coli* promotes the formation of a biofilm matrix in this bacteria.

### Positive selection genes include outer membrane permeability and efflux pumps

Our study succeeded in retrieving the outer membrane porin genes, *ompC* and *ompA*. Porins are beta barrel proteins that serve as channels in the cellular membrane, allowing molecules to pass through. The outer membrane porins are well-known to be under positive selection in bacteria^8,9,30–33^. The *ompA* and *ompC* genes are involved in the maintenance of membrane integrity^34^. Therefore, a mutation in these genes or their deletion can lead to impaired membrane integrity and therefore an increase in the intracellular diffusion of antibiotics. In addition, *ompC* plays a role in antibiotic transport. In fact, outer membrane porins facilitate the passage of small hydrophilic antibiotics, such as β-lactams, as well as tetracycline, chloramphenicol, and fluoroquinolones through the outer membrane^35^. Therefore, a reduction in their capacity develops antibiotic resistance. Also, the ompA gene interacts with the efflux pump systems of the inner membrane (such as major facilitator superfamily (MFS) efflux pumps) and effluxes antibiotics out of the bacterial cell from the periplasmic space^36^.

### Bacterial persistence is used by one gene

Persistence refers to bacteria's strategy of becoming transiently dormant (i.e. temporarily inactive) when they encounter antibiotics. This is because many antibiotics target the actively growing bacteria, and thus persistence allows bacteria to survive the deleterious effect of antibiotics^37,38^. In our study, we found the *hipA* gene to be under positive selection. The *hipA* gene belongs to hipAB, a toxin-antitoxin (TA) module. While many (TA) modules have been linked to the persistent phenotype, hipAB is the first among the TA modules which has been associated with this phenotype. In the hipAB toxin-antitoxin module, the *hipA* gene functions as a toxin, whereas the *hipB* gene functions as an antitoxin, neutralizing the *hipA* toxin. When the concentration of *hipA* is greater than the concentration of *hipB*, the *hipA* gene functions as a toxin, inhibiting cell development and ultimately resulting in dormancy and the creation of persistent bacteria.

### Other targets of positive selection

The selective pressure in bacteria is not limited to antibiotic resistance. Instead, a wide range of selective forces shapes the evolution of bacteria. For example, the host immune system and phages also act as a selective pressure for bacteria, leading to an evolutionary arms race. In our study, we also found a diverse set of functions under positive selection (see Table 1a). For example, we found a class of genes (*bdtT*, *yhaC*, *sbcC*, and *uxuA*), which are involved in “cellular response to DNA damage stimulus “, under positive selection. One might hypothesize that these genes are positively selected due to resisting an attack on DNA from the host immune system, phages, antibiotics, or any other possible forces against bacteria.

### Positive selection genes are core genes, but not essential

Many of the identified genes in our study carry out important functions. Consequently, we hypothesized that they are core genes, which are present in most of the analysed genomes. The genome of *E. coli* is very flexible with a huge difference between the core-genome, the set of genes, which is present in each and every *E. coli* genome, and the pan-genome, which contains all genes across all *E. coli* genomes*.* In our data set, the core-genome has a size of 2783 genes and the pan-genome of 16,582 genes^11^. Out of the 75 genes under positive selection, we found that 33 appear in every genome and another 17 genes belong to 95% or more of the genomes. Considering the genes appearing in at least 95% of genomes as core genes, we have in total 50 core genes under positive selection. On the contrary, we found some genes that appeared only in less than half of the genomes: the restriction enzyme *hsdS,* the rearrangement hotspot element *rhsC,* the putative transposase *insQ,* and transcriptional regulator *dsdC*. However, in summary, the majority of positive selection genes are in the core-genome of *E. coli.*

**Figure 3:**
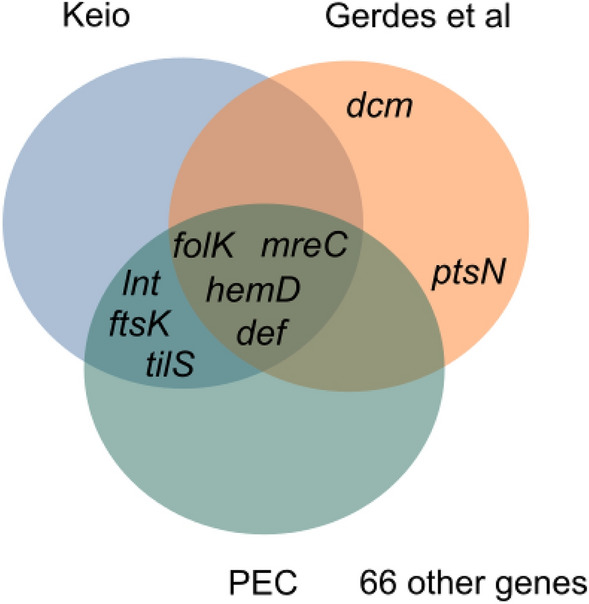
Venn diagram of the essentiality of the positively selected genes based on Keio, Pec, or Gerdes et al. datasets. Nine out of the 75 genes are essential according to at least one of three datasets.

**Figure 4:**
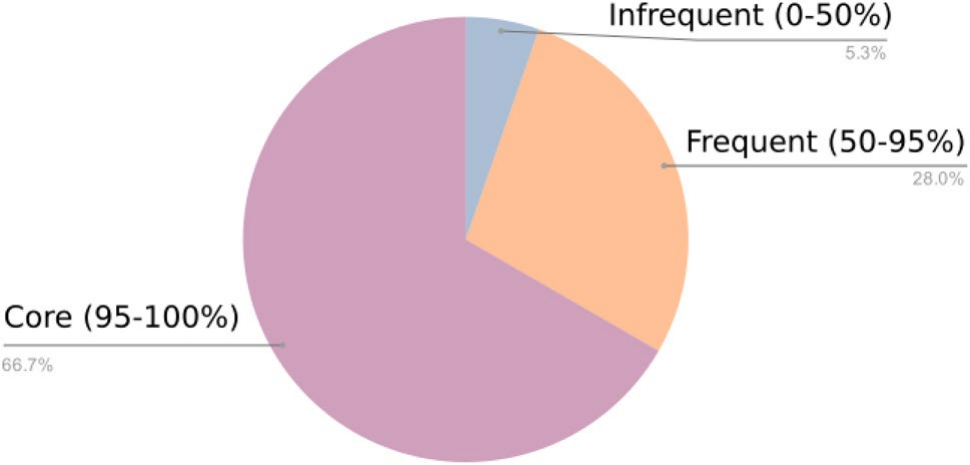
Positive selection and conservation. Frequency of the genes under positive selection across genomes. Most of the genes under positive selection belong to the core-genome.

Next, we asked whether the 75 genes are also essential for growth, which is especially interesting since essential genes are a small subset of core genes^39^. However, we expected that genes under positive selection to be non-essential, as mutations in essential genes may pose a survival risk for the organism. To answer this question, we checked the three datasets of essential genes for *E. coli*: Gerdes et al.^20^, Keio^21^, and PEC^22^. Only nine out of the 75 positively selected genes were identified to be essential according to at least one of these datasets (see Fig. 3). Although most of the positively selected genes are not essential for *E. coli*, we found most of them belong to the core-genome (see Fig. 4). This is because essential genes evolve slowly with few nucleotide substitutions perhaps due to functional constraints required for bacterial survival^40,41^. Therefore, they are under negative selection rather than positive selection.

**Figure 5:**
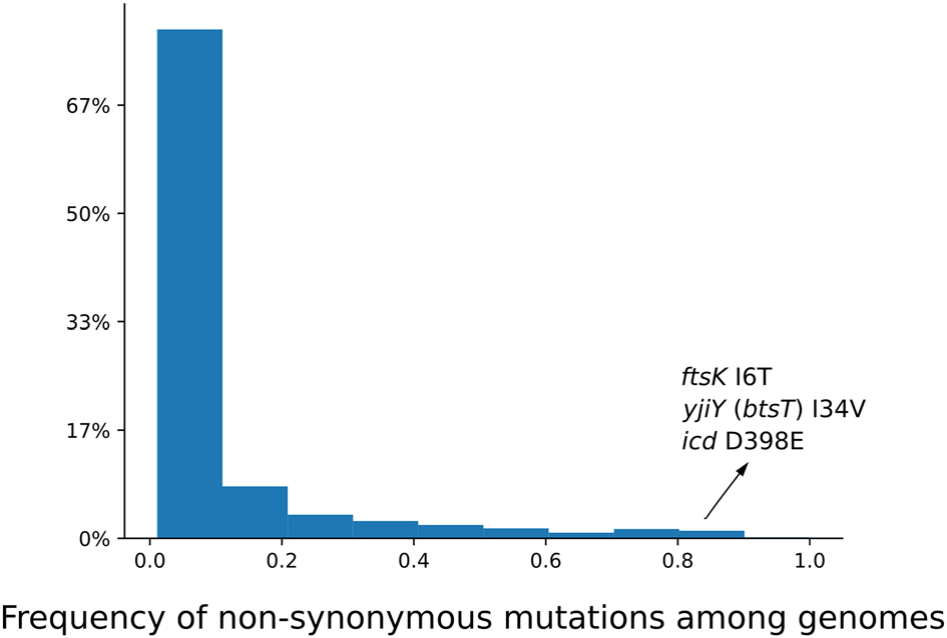
Histogram of the frequency of non-synonymous mutations among genomes. Most of the non-synonymous mutations in core genes are not core mutations.

Positive selection is the result of total mutation load rather than single mutations. Considering that the ratio of substitution rates calculated based on all mutations in the genes, we wondered whether there are some specific frequent non-synonymous mutations, appearing in almost all the genomes, outweigh the effect of rare non-synonymous mutations. In other words, are the mutations responsible for positive selection, core (conserved in at least 95% of the genomes) or not? To answer this question, we considered the 50 positively selected core genes, which are present in at least 95% of the genomes. For each non-synonymous mutation, we recorded whether it is a “core” mutation present in at least 95% of the genomes, in which the gene is present. Figure 5 shows a histogram of these percentages. As shown in Fig. 5, most of the mutations are rare. This means that positive selection is mostly the result of the count of all mutations per gene rather than a count of specific frequent mutations. In other words, it appears to be the mutation burden overall that drives positive selection rather than single frequent mutations with a specific effect. However, it is still interesting to know what the few key conserved core mutations are.

Overall, three out of 2996 non-synonymous mutations have a frequency of more than 95% (see Table 2), which means that they appear to be key mutations. Interestingly, two of these three mutations appeared in all samples (gene = *ftsK*, position = 933,240, allele = C, effect = I6T; gene = *yjiY* (*btsT*), position = 4,591,180, allele = C, effect = I34V). The Ftsk protein is involved in coordinating cell division and chromosome segregation (an essential protein), and the BtsT protein is involved in transporting pyruvate. The other mutation (gene = *icd*, position = 1,196,316, allele = A, effect = D398E) appeared in 88 out of 92 samples. This mutation is inside the *icd* gene, which is one of the seven housekeeping genes used for MLST typing of *E. coli*, and it is a member of the isocitrate and isopropylmalate dehydrogenases family. The core mutation in this gene with the effect of D398E is close to binding sites at positions 391 and 395, and therefore, it is likely of high effect.

**Table 2:** Core non-synonymous mutations among positively selected genes (Frequency > 95%). Freq. refers to the frequency of mutations among our 92 samples.

Gene	Position	Allele	Effect	Freq.	Function
*ftsK*	933240	C	I6T	92 (100%)	Cell division DNA translocase
*yjiY* (*btsT*)	4591180	C	I34V	92 (100%)	Pyruvate: H+ symporter
*icd*	1196316	A	D398E	88 (96%)	Isocitrate dehydrogenase

### Some sites under positive selection correlate with antibiotic resistance

Next, we investigated how positive selection correlates to antibiotic resistance. For each of the 92 genomes, we obtained antibiotic resistance values against 20 widely used antibiotics covering all major classes of antibiotics (for details see^11,42^). We associated the presence of non-synonymous mutations in the positively selected sites of core genes with evidence of antibiotic resistance (*ydhQ*, *dgcE* (*yegE*), *ompC*, *hipA*, *ompA*, and *yfaL*) to the antibiotic values of these 20 antibiotics. Interestingly, we found a correlation between the presence of non-synonymous mutations in some positively selected sites and an increase in antibiotic resistance ([*ompC* A231 & Ceftazidime *p* value: 0.03], [*ompC* G309 & Cefepime, *p* value: 0.01], [*ompC* G309 & Cephalotin,, *p* value: 0.02], [*hipA* I104 & Cefotaxime,, *p* value: 0.03], [*hipA* K290 & Cefotaxime, *p* value: 0.05], [*hipA* G431 & Imipenem, *p* value: 0.03], [*hipA* G431 & Nalidixic acid, *p* value: 0.03]), see Table 3.

**Table 3:** Positive selection and antibiotic resistance. (a) Genes. (b) Mutations.

**(a) For positive selection, our data give the gene’s *p* value and prev. study shows whether the gene appeared in other studies. For genes, core indicates whether a gene is a core gene appearing in at least 95% of genomes and ess. indicates essentiality. The column antibiotic resistance lists resistance functions from literature and whether the gene had antibiotic resistance associated mutations in our data (see B).**						
Gene	Positive selection		Gene		Antibiotic resistance	
	Our data	Prev. study	Core	Ess	Our data	Antibiotics resistance Function
*ompC*	1.57 * 10E−32	Yes	Yes	No	Yes	Membrane permeability
*yegE* (*yfaL*)	5.87 * 10E−19	Yes	Yes	No	No	Biofilm formation
*ycgV*	4.47 * 10E−18	Yes	No	No	No	Biofilm formation
*lsrA*	2.16 * 10E−12	No	No	No	No	Biofilm formation
*ompA*	1.74 * 10E−6	Yes	Yes	No	No	Membrane permeability
*hipA*	1.06 * 10E−5	No	Yes	No	Yes	Bacterial persistence
*ydhQ*	1.06 * 10E−5	No	Yes	No	No	Biofilm formation
*dgcE*	1.06 * 10E−5	No	Yes	No	No	Biofilm formation

**(b) Freq. refers to the frequency of non-synonymous mutations in the site.**						
Gene	Site	Freq	Antibiotic		*P* value	
*ompC*	A231	15	Ceftazidime		0.03	
*ompC*	G309	32	Cefepime		0.01	
			Cephalotin		0.02	
*hipA*	I104	38	Cefotaxime		0.03	
*hipA*	K290	25	Cefotaxime		0.05	
*hipA*	G431	13	Imipenem		0.03	
			Nalidixic acid		0.03	

### Positive selection in the context of 3D structure

**Figure 6:**
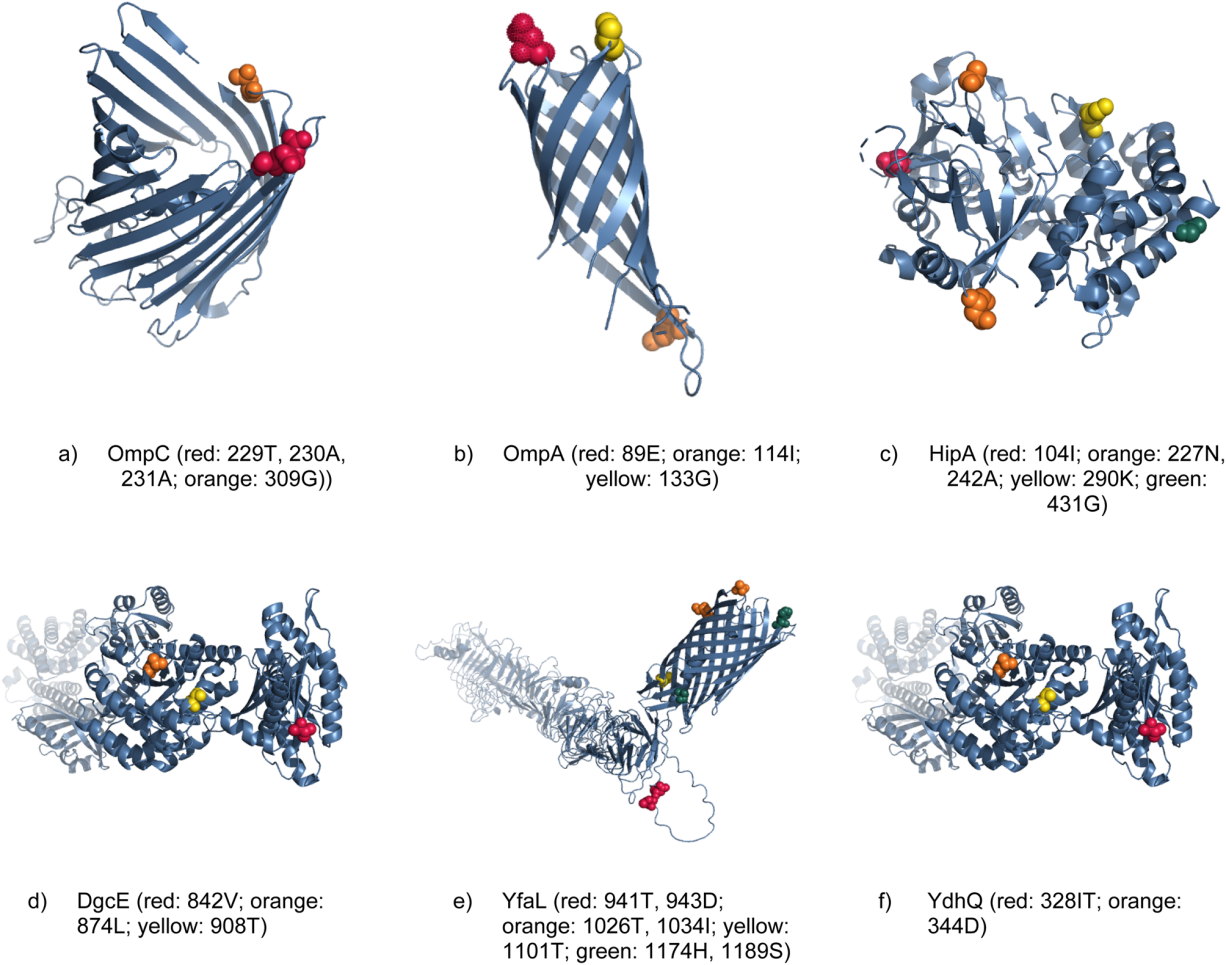
The 3d protein structure of six positively selected core genes with a possible link to antibiotic resistance. The positively selected sites with a probability greater than 0.95 are shown as red/orange/yellow/green spheres.

With the growth of the PDB database^23^ and the recent advent of the Alphafold^24^ database of predicted structures, it is possible to investigate the location of mutations in 3D structures. We hypothesized that the positively selected in the core genes with evidence of antibiotic resistance are located on the surface of proteins. Thus, we obtained structures for the core genes under positive selection that are known to be involved in antibiotic resistance from PDB where possible or from Alphafold. We mapped the mutations onto these structures. Interestingly, we found that these sites are located on the surface of the proteins, where they may play a role in interaction with other proteins or ligands (see Fig. 6). This was especially interesting for beta barrel porins, *ompC* and *ompA*, which are well-known for playing a key role in uptaking antibiotic compounds. A mutation on the rim of the porins possibly interferes with uptaking antibiotic compounds.

## Discussion

### Positive selection complements direct genome-wide association studies with antibiotic resistance

In our previous work^11^, we analysed the core- and pan-genome of the 92 wastewater *E. coli* genomes, which we used in this study. There, we correlated the presence and absence of genes in the genomes against antibiotic resistance levels for 20 antibiotics. Our study revealed a number of genes with a possible link to antibiotic resistance. Later, we extended this line of work and considered the presence and absence of mutations instead of the presence and absence of genes^42^. In that study^42^, we correlated mutations against a phenotype of fluoroquinolone resistance and pinpointed known and novel resistance mechanisms. The latter included the biofilm gene *bdcA*^42^. Both studies relied on antibiotic resistance data. In contrast, the identification of positively selected genes in this study is purely based on an analysis of mutation patterns and independent of antibiotic resistance labels. However, among the positively selected genes, there are some genes that are known to be involved in antibiotic resistance. To shed more light on these genes, we investigated the positively selected sites within these genes with respect to the 3d structure of their protein and checked their association with antibiotic resistance labels. We believe that this approach is powerful and generates novel hypotheses on the emergence and mechanism of antibiotic resistance.

Previous work^8–10^ also identified positively selected genes in *E. coli *independent of any phenotypic labels. However, all of the previous studies were limited to small datasets of less than 10 genomes. We believe that our larger data set of 92 genomes and its genomic diversity originating from a wastewater treatment plant lead to a larger set of identified genes and good overlap with the three previously published studies.

### Model for positive selection

The model for positive selection we used is simple yet powerful. Our study of conservation of mutations driving positive selection supports the choice of a simple count of substitutions. Although there are other models to determine positive selection, these methods are mostly based on haplotype analysis and suitable to detect positive selection in humans and other eukaryotes rather than bacteria^43^. This is because genetic diversity in bacteria is not due to mating and homologous recombination among isolates, but rather clonal expansion.

### Positive selection and antibiotic resistance

It is an interesting question whether specific mutations drive antibiotic resistance or rather an accumulated mutation burden in a gene. An example for the former is *gyrA*, with specific known mutations which lead to quinolone resistance. However, in this study, we found that such a clear relationship between mutation and function appears to be the exception. Only three of 2996 mutations were highly conserved across all genomes. This supports the conclusion that it may be easier for bacteria to evade antibiotics by changing the phenotype through multiple mutations at the gene level rather than only a single mutation.

Another important aspect of our study is the fact that an analysis of positive selection identifies genes that can relate to antibiotic resistance in the first place. However, it should be noted that antibiotic resistance can also happen naturally due to “intrinsic antibiotic resistance” by normal genes. Generally, bacteria might select for many different functions increasing their evolutionary advantage. We found that eight out of 75 positively selected genes can be linked to antibiotic resistance. This supports the conclusion that antibiotic resistance is an important optimization target for the investigated bacteria. It leaves open whether the 67 remaining genes are selected for different reasons or whether they are not yet known to relate to antibiotic resistance. The investigated samples are from a wastewater treatment plant. It will be interesting to investigate how strongly this environment forces bacteria to select for antibiotic resistance.

## Conclusion

Antibiotic resistance is on the rise, and understanding the factors that drive its emergence will play a key role in fighting it. Here, we have demonstrated that it is possible to pinpoint mutations linked to antibiotic resistance starting from genome data only. Our base was a highly diverse set of 92 *E. coli* genomes from a wastewater treatment plant. We identified 75 genes under positive selection by normalising non-synonymous mutations against an overall mutation burden. We showed that many of these genes have a known function relevant for antibiotic resistance such as biofilm formation, reduction of outer membrane permeability, efflux pumps, and bacterial persistence. We found that two-thirds of the genes belong to the core-genome, which means that they appear in nearly all of the investigated samples. However, the majority of the 75 genes (66) are not essential according to at least one of three essentiality studies. The concept of core genes can be transferred to the level of mutations. We investigated whether the mutations under positive selection are core mutations, i.e. they are conserved across most of the samples. We found this mostly not to be the case, which means that positive selection rarely develops highly specific mutations, but rather operates by increasing the mutation burden on the gene level. Next, we looked into six positively selected core genes with the evidence of antibiotic resistance: Two of them harbour mutations, in five sites, which correlate with antibiotic resistance. Finally, we investigated the structural role, which these mutations may play and found that they are accessible at the surface of their proteins in specific locations, which may indicate that they play a direct functional or structural role.

Overall, positive selection analysis on a large set of diverse genomes uncovers known and novel genes and mutations, which drive the emergence of antibiotic resistance.

## Supplementary Information

Supplementary Information.
