# Supplementary material for: A genome-wide scan of wastewater E. coli for genes under positive selection: focusing on mechanisms of antibiotic resistance
Source: Sci Rep. 2022 May 16;12:8037. doi: 10.1038/s41598-022-11432-0 (PMC9110714; doi:10.1038/s41598-022-11432-0)
Supplement: Supplementary file 1 — Supplementary Information. [file 41598_2022_11432_MOESM1_ESM.docx]

| **Supplementary Table S1.** Genes that show evidence of positive selection (Bonferroni-corrected p-value threshold of 1.34×10E-5). Freq. refers to the frequency of a gene among our 92 samples. The dN/dS (ω) column shows the ratio of non-synonymous (dN) to synonymous (dS) substitutions for a gene. | | | | |
| --- | --- | --- | --- | --- |
| **Gene** | **Freq.** | **P-value** | **dN/dS (ω)** | **Description** |
| *hemX* | 92 | 7.32 * 10E-128 | 21.93878 | Uroporphyrinogen III methylase |
| *hisC* | 92 | 3.06 * 10E-42 | 5.23142 | Histidinol-phosphate aminotransferase |
| *gspK* | 87 | 6.81 * 10E-38 | 7.92958 | Type II secretion system protein |
| *ompC* | 90 | 1.57 * 10E-32 | 5.28227 | Outer membrane porin C |
| *ydfJ* | 86 | 1.81 * 10E-25 | 21.38778 | Putative transporter |
| *yhaC* | 54 | 2.07 * 10E-23 | 5.51939 | Uncharacterized protein |
| *paaF* | 65 | 4.15 * 10E-21 | 40.8696 | Putative 2,3-dehydroadipyl-CoA hydratase |
| *elaD* | 74 | 8.26 * 10E-20 | 13.04257 | Deubiquitinating protease |
| *folK* | 92 | 1.34 * 10E-19 | 5.22309 | 2-amino-4-hydroxy-6-hydroxymethyldihydropteridine diphosphokinase |
| *yfaL* | 91 | 5.87 * 10E-19 | 6.70763 | Putative autotransporter adhesin |
| *ycgV* | 71 | 4.47 * 10E-18 | 5.49057 | Putative autotransporter adhesin |
| *lacZ* | 92 | 1.52 * 10E-16 | 3.92836 | β-galactosidase |
| *rhsC* | 20 | 2.27 * 10E-16 | 4.70745 | Rhs element protein |
| *flgK* | 92 | 4.14 * 10E-16 | 7.17042 | Flagellar hook-filament junction protein 1 |
| *ydiU* | 92 | 1.02 * 10E-12 | 7.22815 | Protein adenylyltransferase |
| *ptsP* | 92 | 1.06 * 10E-12 | 3.69025 | Protein adenylyltransferase |
| *lsrA* | 78 | 2.16 * 10E-12 | 6.26522 | Autoinducer-2 ABC transporter ATP binding subunit |
| *argE* | 92 | 2.23 * 10E-12 | 18.77897 | Acetylornithine deacetylase |
| *agaC* | 91 | 7.70 * 10E-12 | 7.73671 | Galactosamine-specific PTS enzyme IIC component |
| *yhdP* | 92 | 2.51 * 10E-11 | 4.62862 | Phospholipid transport factor |
| *rarD* | 92 | 3.25 * 10E-11 | 5.96233 | Inner membrane putative transporter protein |
| *def* | 92 | 3.85 * 10E-11 | 7.55998 | Peptide deformylase |
| *insQ* | 29 | 6.10 * 10E-11 | 6.37264 | Putative insertion element transposase |
| *lnt* | 92 | 7.99 * 10E-11 | 6.141 | Apolipoprotein N-acyltransferase |
| *dcm* | 92 | 1.09 * 10E-10 | 7.48329 | DNA-cytosine methyltransferase |
| *entF* | 92 | 1.93 * 10E-10 | 8.81407 | Apo-serine activating enzyme |
| *ptsN* | 92 | 2.15 * 10E-10 | 3.02599 | Phosphotransferase system enzyme IIA |
| *glcB* | 78 | 3.31 * 10E-10 | 6.57713 | Malate synthase G |
| *gspL* | 89 | 2.65 * 10E-9 | 3.06889 | Type II secretion system protein |
| *dsdC* | 37 | 6.07 * 10E-9 | 12.30115 | DNA-binding transcriptional dual regulator |
| *hyfB* | 78 | 7.56 * 10E-9 | 6.35196 | Hydrogenase 4 component B |
| *pphC* | 90 | 2.23 * 10E-8 | 7.54815 | Protein-serine/threonine phosphatase |
| *deaD* | 92 | 3.19 * 10E-8 | 3.11358 | ATP-dependent RNA helicase |
| *yjiR* | 62 | 3.67 * 10E-8 | 3.47378 | Fused putative DNA-binding transcriptional regulator/putative aminotransferase |
| *uxuA* | 92 | 3.71 * 10E-8 | 14.90417 | D-mannonate dehydratase |
| *icd* | 92 | 6.96 * 10E-8 | 15.04868 | Isocitrate dehydrogenase |
| *cpdB* | 92 | 1.03 * 10E-7 | 4.09655 | 2'3' cyclic nucleotide phosphodiesterase/3' nucleotidase |
| *fruA* | 92 | 1.24 * 10E-7 | 4.62264 | Fructose-specific PTS multiphosphoryl transfer protein |
| *btsT* | 92 | 1.47 * 10E-7 | 11.5251 | Pyruvate: H+ symporter |
| *mreC* | 92 | 1.49 * 10E-7 | 7.70834 | Cell shape determining protein |
| *yphH* | 90 | 1.60 * 10E-7 | 12.08929 | Putative DNA-binding transcriptional regulator |
| *tmcA* | 92 | 1.82 * 10E-7 | 3.30687 | tRNAMet cytidine acetyltransferase |
| *ynbB* | 77 | 2.80 * 10E-7 | 5.30319 | Putative CDP-diglyceride synthase |
| *ftsK* | 92 | 2.80 * 10E-7 | 6.06923 | Cell division DNA translocase |
| *gspC* | 84 | 2.85 * 10E-7 | 3.5465 | Type II secretion system protein |
| *bisC* | 92 | 3.06 * 10E-7 | 4.1772 | Biotin sulfoxide reductase |
| *sbcC* | 92 | 4.01 * 10E-7 | 4.10683 | ATP-dependent structure-specific DNA nuclease |
| *ydbH* | 89 | 4.66 * 10E-7 | 5.56828 | Autotransporter (AT) family protein |
| *yphG* | 89 | 5.20 * 10E-7 | 3.71438 | DUF5107 domain-containing protein |
| *betA* | 82 | 5.41 * 10E-7 | 18.2539 | Choline dehydrogenase |
| *rhaB* | 92 | 5.74 * 10E-7 | 3.82552 | Rhamnulokinase |
| *wzxC* | 80 | 7.99 * 10E-7 | 3.96171 | Colanic acid repeat unit flippase |
| *ynbC* | 83 | 1.09 * 10E-6 | 2.70306 | Hydrolase/methyltransferase domain-containing protein |
| *nanS* | 70 | 1.09 * 10E-6 | 5.30055 | N-acetyl-9-O-acetylneuraminate esterase |
| *dgcE* | 89 | 1.13 * 10E-6 | 3.13864 | Putative diguanylate cyclase |
| *hemD* | 92 | 1.41 * 10E-6 | 12.32034 | Uroporphyrinogen-III synthase |
| *dtpD* | 92 | 1.46 * 10E-6 | 24.39876 | Dipeptide: H+ symporter |
| *yciW* | 90 | 1.47 * 10E-6 | 4.10337 | Putative oxidoreductase |
| *ompA* | 92 | 1.74 * 10E-6 | 4.05251 | Outer membrane protein A |
| *tilS* | 92 | 1.87 * 10E-6 | 3.57307 | tRNAIle-lysidine synthetase |
| *alkA* | 91 | 1.91 * 10E-6 | 4.22584 | DNA-3-methyladenine glycosylase 2 |
| *cadC* | 85 | 2.71 * 10E-6 | 3.34191 | DNA-binding transcriptional activator |
| *uidC* | 91 | 3.14 * 10E-6 | 12.50101 | Outer membrane porin family protein |
| *abgB* | 72 | 3.31 * 10E-6 | 12.60796 | p-aminobenzoyl-glutamate hydrolase subunit B |
| *bglB* | 76 | 4.08 * 10E-6 | 3.50765 | 6-phospho-β-glucosidase B |
| *yhaV* | 87 | 4.64 * 10E-6 | 20.25387 | Ribosome-dependent mRNA interferase toxin |
| *hsdS* | 10 | 5.29 * 10E-6 | 27.01265 | Type I restriction enzyme EcoKI specificity protein |
| *yhiN* | 91 | 6.27 * 10E-6 | 3.36877 | Putative oxidoreductase |
| *yegH* | 91 | 7.07 * 10E-6 | 4.20122 | Inner membrane protein |
| *mhpA* | 83 | 7.14 * 10E-6 | 3.85192 | 3-(3-hydroxyphenyl)propanoate hydroxylase |
| *hipA* | 89 | 1.06 * 10E-5 | 4.42652 | Serine/threonine-protein kinase toxin |
| *alkB* | 92 | 1.09 * 10E-5 | 7.56112 | DNA oxidative demethylase |
| *yegD* | 92 | 1.10 * 10E-5 | 2.88176 | HSP70 family protein |
| *ydhQ* | 91 | 1.11 * 10E-5 | 4.94002 | Putative adhesin-related protein |
| *yhhS* | 88 | 1.31 * 10E-5 | 7.56769 | Putative transporter |
